# Advances in Cathepsin S Inhibition: Challenges and Breakthroughs in Drug Development

**DOI:** 10.3390/pathophysiology31030035

**Published:** 2024-09-03

**Authors:** Temitope A. Ajani, Zandisiwe E. Magwebu, Chesa G. Chauke, Kenechukwu Obikeze

**Affiliations:** 1 School of Pharmacy, University of the Western Cape, Cape Town 7535, South Africa; temitopeajani@gmail.com; 2 South African Medical Research Council, Primate Unit and Delft Animal Centre (PUDAC), Cape Town 7100, South Africa; zandisiwe.magwebu@mrc.ac.za (Z.E.M.); chesa.chauke@mrc.ac.za (C.G.C.)

**Keywords:** Cathepsin S, proteolytic enzyme, cathepsin S inhibitor, specificity, clinical trial

## Abstract

Cathepsin S (CatS) is a proteolytic enzyme and a member of the cysteine protease family of proteolytic enzymes. Cathepsins S, K, and L are particularly similar in terms of their amino acid sequences and interactions with substrates, and this has made it difficult to develop inhibitors with specificity for either CatS, CatK, or CatL. The involvement of CatS in various disease pathophysiologies (autoimmune disorders, cardiovascular diseases, cancer, etc.) has made it a very important target in drug development. Efforts have been made since the early 1990s to develop a specific CatS inhibitor without any major success. Following many failed efforts to develop an inhibitor for CatS, it was discovered that interactions with the amino acid residues at the S2 and S3 pockets of CatS are critical for the identification of CatS-specific inhibitors. Amino acid residues at these pockets have been the target of recent research focused on developing a non-covalent, reversible, and specific CatS inhibitor. Methods applied in the identification of CatS inhibitors include molecular modeling, in-vitro screening, and in-vivo studies. The molecular modeling process has proven to be very successful in the identification of CatS-specific inhibitors, with R05459072 (Hoffmann-La Roche) and LY3000328 (Eli Lilly Company) which has completed phase 1 clinical trials. CatS inhibitors identified from 2011 to 2023 with promising prospects are discussed in this article.

## 1. Introduction

Cathepsins are lysosomal proteolytic enzymes that belong to the papain family. The human cathepsin family consists of 15 members which are classified as serine (Cathepsin A and G), aspartate (Cathepsin D and E), and cysteine (B, C, F, H, K, L, O, S, V, W, and Z) proteases based on the amino acid residue present at the active site/catalytic center [1,2]. The cysteine cathepsins are made in the endoplasmic reticulum as preprocathepsin and stored in the acidic environment of the lysosome where they are cleaved to their mature form. Cathepsin S (CatS) is a member of the cysteine cathepsin family with the unique characteristics of being an active catalyst within a wide pH range (5–7.5) [3]. Apart from its activities within this pH range, its involvement in the pathophysiology of several diseases has been reported. Increases in CatS expression have been reported in oral cancer [4], prostate cancer [5], lung cancer [6], diabetes mellitus [7], atherosclerosis [8], hypertension [9], myocardial infarction [10], obesity [11], autoimmune diseases [12,13], and many more. The establishment of its involvement in the pathophysiology of these diseases has made CatS an enzyme of interest in disease management. Pharmacological inhibition of this cathepsin has been demonstrated to be beneficial in the management of abdominal aortic aneurysm [14], inflammation [15], atherosclerosis [16], cancer [17], myocardial ischemia and reperfusion injury [18], autoimmune diseases [19], and other diseases. Several attempts have been made to identify an inhibitor for this enzyme, but only RO5459072 by Hoffman-La Roche has passed stage II clinical trials. However, Leroy and Thurairatnam [20] and Lee-Dutra and Wiener [21] have reviewed and documented the effort made by different researchers to identify CatS inhibitor between the period when the crystal structure of CatS was elucidated in 1998 and 2010 without any major breakthroughs. After their review in 2011, no other review article known to us has been published on CatS inhibitors. With the establishment of its involvement in other diseases that were not known prior to the previous review, it is important to document the new developments in identifying inhibitors for this proteolytic enzyme. The purpose of this article is to provide a summary of the published work towards identifying new CatS enzyme inhibitors from the end date of the previous review (2011) until 2023.

## 2. Methodology

Articles on CatS and its inhibitors were searched for across the Web of Science (WOS) website (https://www.webofscience.com/wos/woscc/basic-search, accessed on 14 April 2024) and Scopus website (https://www.scopus.com, accessed on 14 April 2024) from 2012 to 2023 using keywords including “*Cathepsin S*” or “*Cat s*” or “*Cathepsin S inhibitor*” or “*Cathepsin S inhibitors*” or “*Cat s inhibitor*” or “*Cat s inhibitors*” to generate a total of 3118 articles from both Scopus and WOS. The bibliometric tool in the R program was used to merge the files and removed 532 duplicated articles. This trimmed the number of articles to 2586 articles. These articles were further subjected to more filters by looking for articles that had Cathepsin S and its inhibitor in their title or abstract and this left us with 290 articles which were then selected, and the specific articles reporting on the synthesis and/or isolation of CatS enzyme inhibitors are reported in this review.

## 3. Cathepsin S

**Figure 1:**
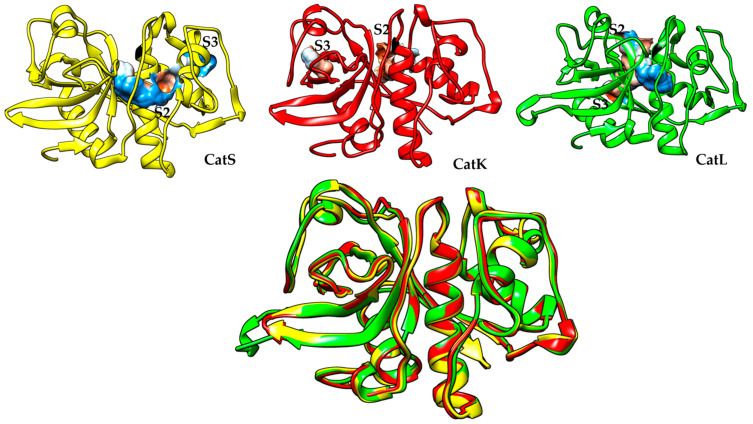
The 3D structure of CatS 3N3G (Yellow), CatK 1MEM (Red), and CatL 3HHA (Green) superimposed together. The S2 and S3 pocket of each macromolecule is marked. (Source: https://www.rcsb.org, accessed on 15 August 2024).

CatS is a proteolytic enzyme that acts as an endopeptidase in the cleavage of proteins into peptides. It is responsible for the cleaving of the extracellular matrix, collagen, proteoglycans, and elastin. The mature enzyme is made up of 217 amino acid residues and is expressed by most immune cells including macrophages, vascular smooth muscle cells, epithelial cells, endothelial cells, and neutrophils. Its expression is commensurate with the expression of the endogenous inhibitor cystatin C, except in pathological conditions where CatS is expressed or secreted in greater quantities than the endogenous inhibitor. This imbalance in the expression of the enzyme and its inhibitor has been linked to the progression of many inflammatory diseases [22,23]. The amino acid residues that constitute the catalytic triad for both CatK and CatS are Cysteine (Cys), Histidine (His), and Asparagine (Asn). In fact, CatS, L, and K contain similar amino acid residues for more than 57% of the amino acid sequences [24,25], but despite the significant similarity, there are significant differences in their biological actions. In Figure 1 below, the structure of CatS, CatK, and CatL are superimposed on top of each other, and it is difficult to spot the difference because of their structural similarities. The close similarity in the amino acid sequence between these cathepsins has made it difficult to identify or develop a reversible, non-covalent, and specific inhibitor for each of these cathepsins. In line with this, enzyme specificity is a factor to be seriously considered in developing an inhibitor for any of these cathepsins.

The amino acid residues that constitute the catalytic triad for CatS are Cys25, His159, and Asn175 [26]; for CatK they are Cys25, His162, and Asn182 [27]; and for CatL they are Cys25 and H163 and Asn179 [28]. Cys25 is located in the active sites of all the isomers, but CatS is distinct in the residues at the S1′ to S3 sites, which determines the binding specificity of the CatS enzyme. The S3 amino acid residues are Gly62, Asn 63, Lys 64, Gly 68, Gly 69, and Phe 70. The S2 amino acid residues are Phe 70, Gly 137, Val 162, Gly 165, and Phe 211, while S1 amino acid residues include Gly23, Gly68, Asn67, and Cys22 linked to Cys66 with a disulfide bridge. The S1′ binding pocket has amino acids in both the proximal and distal regions. Ala140, Arg141, Asn163, His 164, and Trp186 are in the proximal region, and the His142, Pro143, and Phe146 amino acid residues are in the distal region. These pockets and their amino acid residues constitute the amino acid residues responsible for the specificity of CatS enzymes compared with other cathepsin isoenzymes [17,26]. The point of difference between these enzymes lies in the shape and the amino acid residue at the S2 and S3 pockets [26,29,30]. CatS and CatK specifically have overlapping substrate specificity. When they are overexpressed or dysregulated, they are found in other compartments like the cytoplasm, endosome, and the mitochondria that are outside the lysosome. In this form, they are involved in cleaving of extracellular matrix constituents. CatK inhibition is connected to the treatment of osteoporosis and bone metastasis [27].

An early focus of research to identify an inhibitor for CatS was the identification of a potent inhibitor, but this did not turn out well as most of the inhibitors developed this way lacked specificity for CatS and interacted with or inhibited other cysteine cathepsins. For example, Pattersen et al. reported on a search for CatS inhibitors focusing on the potency of the inhibitor which resulted in the development of a CatK inhibitor [31]. As such, the selectivity of the compound became more important than the potency of the compound in the evaluation of compounds being considered as CatS inhibitors. Most of the recent research on this subject now focuses on the synthesis and identification of potent and specific CatS inhibitors. In some research, potent compounds identified in the past have now been modified to become more specific CatS inhibitors.

The potency of an inhibitor is mainly determined by the type of interaction that occurs between the amino acids at the active site, especially Cys25, the substrate groove, and the inhibitor. This interaction can be a noncovalent or covalent irreversible interaction or a covalent reversible interaction, with reversible interactions being the preferred interaction in the case of drug development. The S2 and S3 pocket amino acids are mainly responsible for compound selectivity, while covalent bonding (reversible or irreversible) to cysteine at the active site of CatS enzyme is important for potency [32]. However, to identify a highly selective inhibitor with great potency, interaction with the S2 and S3 pockets is of paramount importance. Other amino acids that are not at the S2 or S3 pocket also play important roles, for example, the position of the Phe 211, Gly 137, and Val 162 are responsible for enhancing pocket plasticity, restricting the entry of compounds, and making the pocket in the CatS enzyme deeper. This determines how an inhibitor interacts with the enzyme [33].

## 4. Development of CatS Inhibitors

### 4.1. CatS Inhibitors from Proline Analogs with a Vinyl Sulfone Group

**Figure 2:**
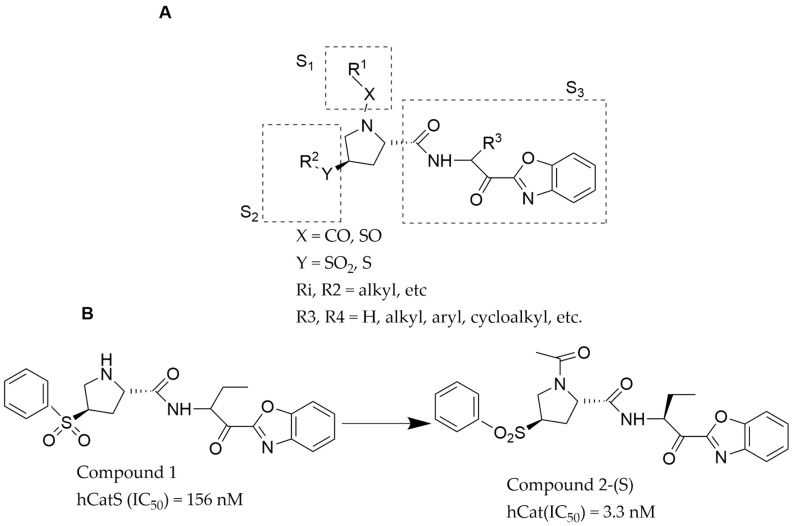
The chemical structure of compounds **1** (**A**), **2**, and **2**-(S) (**B**) [34].

With the insight into the amino acid residue that confers specificity on CatS and successes recorded in past proline analog projects [21], Kim and Jeon [34] screened a library of proline-based compounds to identify a hit compound **1** with an IC_50_ of 156 nM for CatS (Figure 2). This potentially potent compound was then modified to be specific for CatS by adding a moiety to allow it to interact with the amino acid residues at the S2 and S3 pockets of the CatS enzyme. Compound **1** was investigated in-vitro for specificity for CatS [28] and then modified (via methylation, nucleophilic substitution, oxidation, derivatization, and hydrolysis) without altering the moiety responsible for its specificity until a more potent compound was identified. After each modification, the resulting compounds were tested to confirm that they maintained their specificity, and the potency was compared with compound **1**’s potency. A series of compounds were synthesized during the process but compound **2** (Figure 2A) proved to be the most potent.

The chemical structure of compound **2** was segmented into 3 different components S1, S2, and S3 (Figure 2A) to explore the structure-activity relationship (SAR) of the compound. Substituting a methyl group at the S1 moiety increased the potency of compound **2**, while substitutions at the S2 moiety had no effect on the potency; however, substitution at the S3 moiety reduced the CatS inhibitory activity of compound **2**. Five (5) compounds with a high potency developed from substitutions at the S1 and S2 moieties of compound **2** were tested in-vitro with CatK and CatB and showed no activity and selectivity for both enzymes. Therefore, the most important moiety for improving the activities and potency of compound **2** is the S1 moiety (Figure 2B). The methylation of the S1 moiety yielded compound **2**-(S) with an IC_50_ of 3.3 nM and suitable pharmacokinetics properties, fitting for a lead compound in drug development [34].

**Figure 3:**
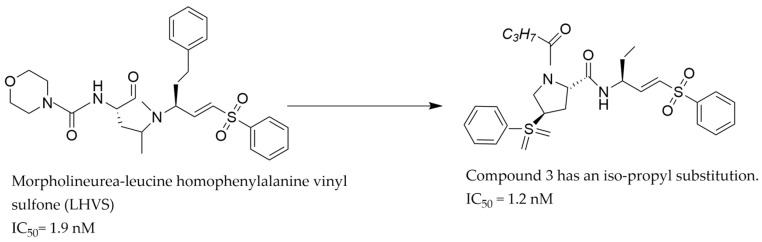
The chemical structure of LHVS and compound **3** [35].

After the successful identification of the proline-based compound **2**-(S) with good potency, CatS selectivity, and suitable pharmacokinetic properties (bioavailability, half-life, molecular weight, and solubility), Kim and Jeon [35] expanded their search for CatS inhibitors by considering the proline derivative since they had great success from their earlier research on proline derivatives [33]. In this case, they were interested in modifying a known irreversible non-selective CatS inhibitor, Morpholineurea-leucine homophenylalanine vinyl sulfone 1 (LHVS) [21,36], into a reversible and specific CatS inhibitor. LHVS was one of the first CatS inhibitors identified as a potent inhibitor but was not specific for CatS as it also inhibits CatK [21]. Through a series of biochemical processes on the precursor compound LHVS, the vinyl sulfone moiety of the compound was retained since this is responsible for its biological activities, but the proline moiety was modified to improve its potency and selectivity. A series of compounds emerged from this modification with IC_50_ values ranging from 1.2 nM to 2.6 nM, which were not superior to LHVS which also had an IC_50_ value within the same range. However, these compounds showed more specificity for CatS than LHVS in-vitro [35]. Specifically, compound **3** (Figure 3) was more potent than LHVS and has iso-propyl and proton substitution at positions R1 and R2 of LHVS, respectively. Apart from the specificity of compound **3** for CatS which was an advancement over LHVS, the type of interaction that exists between compound **3** and CatS was not reported [21,35].

### 4.2. Pyrazole-Based Aryl Alkyne CatS Inhibitor

**Figure 4:**
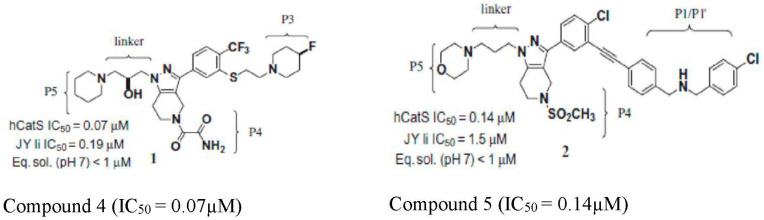
Chemical structure of compound **4** and compound **5** [38].

Identification of CatS inhibitors from pyrazole-based aryl alkynes was a project started by Johnson and Johnson in 2009 with very promising results as a noncovalent inhibitor of CatS [37]. It has been well-reviewed by Lee-Dutra, Wiener [21]. Their earlier research yielded two CatS inhibitors called compounds **4** and **5** (Figure 4) with various advantages and limitations. These compounds interact with the amino acid residues at the S1/S1′ and S4 pockets of the CatS enzyme. The compounds were potent, but the pharmacokinetics properties were not suitable for drug development. The research on these compounds was expanded by Janssen Research and Development [38] to improve upon the strengths and mitigate the limitations of the initial compounds. For example, the limitations of compound **4** include poor oral bioavailability but a good potency with an IC_50_ of 0.07 µM. Compound **5** on the other hand has good pharmacokinetics properties but a lower potency with an IC_50_ of 0.14 µM. The potency of compound **4** and the suitable pharmacokinetics properties of compound **5** became attractive and Wiener and Wickboldt [38]) explored these to develop a compound that interacts with the amino acid residue at the S2 and S3 pocket of CatS, hence developing a more specific and potent CatS inhibitor.

From Figure 4 above, it can be seen that the oxamide (P4) of compound **4**, which is believed to be responsible for its potency, interacts with the amino acid residue at the S4 pocket of the CatS enzyme, and the Alkyne (P1/P1’) of compound **5** interacts with the amino acid residue at the S1/S1′ pocket of the CatS enzyme. Compound **5** with the alkyne side chain was synthesized in a way that favors the substitution at the p4 side chain with different moieties to alter the pharmacokinetics and potency of the compound. When the chemical substituent at P4 on compound **5** was removed, the compound lost its potency. However, substitution with oxamide and urea improved the potency with an IC_50_ of 0.06 µM and 0.09 µM, respectively. Further substitutions of different chemical groups at P4 did not change the potency of compound **5**. Substitution at the P5 on compound **5** did not improve its potency or pharmacokinetic properties. When an amine functional group was inserted at P5, the cellular potency of the compound was eliminated as the amine functional group contributed to the accumulation of the compound **5** in the lysosome but enzymatic potency was not altered, and this agreed with other research on pyrazole-based CatS inhibitors [37,39]. They went on to improve the solubility of the compound as this was a major setback with the earlier compound. However, they did not explore the specificity of the new compound on other members of the cysteine protease enzyme family. In addition, there is no report of the constituent interaction with the S2 and S3 amino acid residue of the CatS enzyme. Therefore, a need exists to explore the interaction of compound **5** with amino acid sequence at the S2 and S3 pockets of the enzyme [38].

### 4.3. Central Cyclic Scaffold-Based CatS Inhibitors

Several successes have been recorded with the nitrile succinimide and the triazole derivatives of CatS inhibitors [40]. Compound C, which is a cyclopentane derivative, was developed by Roche as a CatK inhibitor with modest activity on CatS [41]. Hilpert and Mauser [40], modified compound C to interact with the amino acid residues at the S2 and S3 pockets of CatS by adding a chemical moiety that interacted with these amino acid residues to make the compound more selective for CatS. The R2 constituent of the compound was found to interact with the amino acid residue at the S2 pocket of CatS, which is one of the pockets identified to be responsible for the specificity of the enzyme [28]. Significant emphasis was placed on exploring interactions with the amino acid residues located within the S2 pocket of CatS, aiming to identify a more selective inhibitor for CatS. It was a deliberate action to avoid interactions with CatK, primarily due to the resemblance between the S1 and S3 pockets of both CatK and CatS.

Cyclic moiety was considered as a moiety for this side chain R2 because a metabolic breakdown of the cyclic moiety is slower and the number of rotations possible at this cyclic moiety is limited [40]. Five Cyclic moieties including cyclopentane, pyrrolidines, prolines, homocyclic and heterocyclic scaffolds were considered. Extension into the S2 pocket by the cyclopentane-cyclopropyl methyl sulfone moiety improved the selectivity and potency of the initial compound with over 20,000-fold selectivity for CatS and an IC_50_ of 0.7 nM compared to the IC_50_ of the initial compound for CatS of 925 nM in an in vitro study. The nitrile group forms a reversible covalent bond with Cys25 at the S1 pocket. The cyclopentane moiety shows the most appropriate improvement in potency and selectivity for CatS. Other moieties show no significant improvements in their selectivity and biological activities [40].

### 4.4. LY3000328 CatS Inhibitor

**Figure 5:**
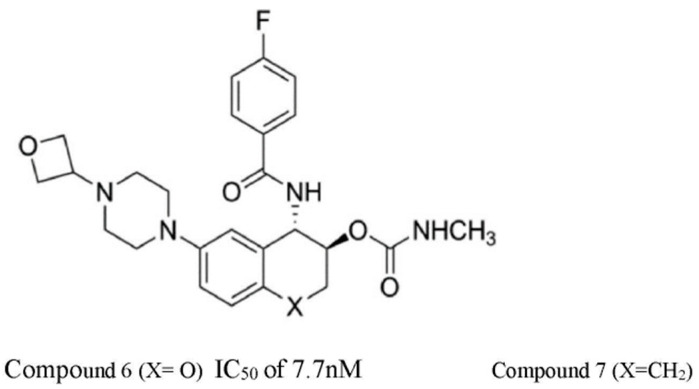
The chemical structure of compounds **6** and **7**. (The difference between compounds **6** and **7** is the substituent at position X) [42].

Eli Lilly, who have a strong research interest in CatS inhibitors through their earlier investigations, revived their search for a CatS inhibitor. They conducted a high throughput screening of a database of 120,000 compounds to identify a hit compound. The hits compound was modified to become a specific CatS non-covalent selective inhibitor [42]. Learning from other previous failed attempts, they paid attention to compounds that interact with the amino acid residue at the S2 and S3 pockets of the enzyme. In a way to avoid an irreversible covalent interaction, compounds that covalently bonded to the Cys25 at the active site of the enzyme were excluded. Their screening yielded several compounds but compound **6** which is named LY3000328 was identified as the most potent and selective for CatS (Figure 5). It interacts with the amino acid residue at the S2 and S3 pockets of the enzyme [42,43].

Compounds **6** and **7** proved to be very potent and selective for cathepsin but compound **6** was more suitable with very good pharmacokinetic properties. The compounds were tested on the CatS enzyme in humans and mice and compound **6** had an IC_50_ of 7.7 nM in humans and 1.67 nM in mice (Table 1). Compound **6** was selected as the most suitable compound for the drug candidate. It was then tested against other cathepsins (B, L, K, and V), and it was proven to be more selective for CatS than the other cathepsins. However, data for the degree or level of specificity were not included in their report [42]. LY3000328 has since been used as an inhibitor of CatS in the disease model. Its inhibitory activities in myocardial/reperfusion injury have been reported [18] and its inhibitory action on abdominal aortic aneurysm has received attention as far as CatS inhibition is concerned [42].

### 4.5. Cathepsin S Inhibitor from Nuclear Magnetic Resonance Fragment Screening

Schade et al. and Merla [44] adopted the NMR fragment screening and crystal structure-aided merging method to identify a CatS specific inhibitor. The method identifies a hit compound for CatS inhibition. Fragment drug discovery is a method that adopts putting together different parts of a drug molecule bit by bit. A hit compound is generated by screening a fragment-based database (FBD). This database normally consists of a fragment of a compound that obeys the rule of three (3) just like Lipinski’s rule of five in drug development. The rule of three simply suggests a small compound that can be included in the library of FBD. The compounds must have a molecular weight of 300 Da or lower, cLogP of three or less, three hydrogen bond acceptors and donors, three rotatable bonds, and a molecular polar surface area of 60 or less. Fragment-based drug discovery has been well-reviewed by Singh and Tam [50] and Kirsch and Hartman [51]. Few drugs developed through this method are already in use and others are in clinical trials. Vemurafenib (treatment of metastatic melanoma), Erdafitinib (treatment of metastatic bladder cancer), Pexidartinib (treatment of tenosynovial giant cell tumor), Sotorasib (non-small cell lung cancer), were all approved in the last 5 years except for Vemurafenib [52,53].

**Figure 6:**
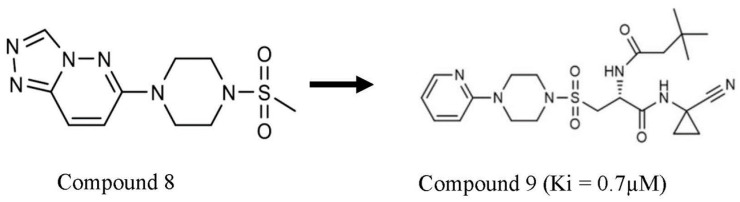
Compound **8** and compound **9**. Structure adapted from Schade, Merla [44].

The method adopted the use of a small fragment of compound targeted at the S2 and S3 pocket of the CatS enzyme. Special attention was given to compounds that are bound by other forms of interaction apart from the covalent interaction. One thousand eight hundred and fifty-eight (1858) small fragment compounds were screened against CatS enzymes. Eighteen hit compounds were identified to interact with either the amino acid residues at the S2 or S3 pockets of CatS. Out of these 18 compounds, only six (6) showed more than 50% selectivity for CatS over other cysteine cathepsin enzymes in an in vitro assay. Three of these compounds interact with the amino acid residues at the S2 pocket of CatS enzyme with a type of interaction that was not seen with other cathepsin family members. Compound **8** (Figure 6) was identified to fit into the S2 pocket, the step thing was identifying fragments or moieties to join to compound **8** that will enhance its interaction with the amino acid residue at the S3 pocket of the enzyme. The moiety that interacts with the S3 pocket and the catalytic center was added to compound **8**. The literature was searched to identify compounds that interact with the amino acid at the catalytic center and S3 pocket of CatS. These compounds were compared with improved compound **8** and they were matched for fragment elongation. The N-(1-cyanocyclopropyl) amide (Petesicatib-RO5459072) which has completed stage 2 clinical trial (Table 2) was compared with compound **8** to design an extension of the compound (Table 2). The sulfonyl group on compound **8** was extended with an N-methyl propanamide to yield a compound **9** that was 100 times better in terms of potency and interaction as compared with compound **8**, with a Ki of 0.7 µM and high specificity for CatS (Table 1). This extension forms the backbone of the hydrogen bond that interacts with the amino acid residue at the S1 pocket of CatS which does not necessarily improve the potency and CatS selectivity for the compound [44].

### 4.6. RO5444101 as Cathepsin S Inhibitor

**Figure 7:**
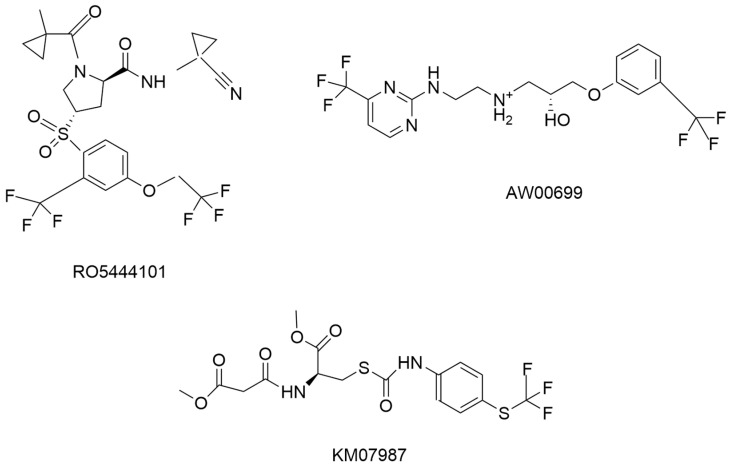
The chemical structure of R05444101, AW00699, and KM07987 [46].

RO5444101 (Figure 7) was first reported by Hoffman La Roche, Basel, Switzerland as a CatS selective inhibitor in the management of atherosclerosis in Apolipoprotein E deficient mice suffering from chronic renal disease [45]. Limited information was provided on the synthesis of this inhibitor except that it was a highly potent and selective CatS inhibitor (interacting with amino acid residues at the S2 pocket) with an IC_50_ of 0.2 nM in humans and IC_50_ of 0.3 nM in the mouse CatS model. It showed good pharmacokinetic properties in the management of atherosclerosis. Additionally, it was tested against other cysteine cathepsin members and was more than 25,000 times more selective for CatS than other members of the cysteine cathepsin family. Figueiredo et al. and Aikawa [45] did not report on its interaction with CatS. The downside of R05444101 is its molecular weight (567.51 Da) and its therapeutic performance attribute (TPA) which needs to be modified before it can be considered as a drug candidate.

Based on the success and limitations recorded with RO5444101 by Roche, Ahmad and Bhagwati [46] explored the modification of RO5444101 to improve the pharmacokinetic properties and potency using a molecular modeling approach. Molecular modeling is a process of drug development that involves the use of a computer model to predict drug pharmacokinetics and pharmacodynamic properties through different types of applications and software [53]. Molecular modeling was adopted in the identification of a hit compound with similar pharmacokinetics and potency to RO5444101. Other CatS inhibitors with a similar structure to RO5444101 were also considered. The protein data bank was searched to identify CatS 3D structure with co-crystalized inhibitor non-covalent bonds. Twenty (20) CatS inhibitor complexes were downloaded but only six (6) complexes of CatS enzymes and inhibitor crystal structures (4P6G, 3N3G, 4P6E, 1MS6, 3N4C, and 2R9M) were identified to have a similar structure to RO5444101 and had IC_50_ values of 7.7 nM, 58 nM, 1290 nM, 19 nM, 9.5 nM, and 1.5 nM, respectively. The ligand (inhibitor) of these structures was aligned with RO5444101 through the flexible ligand alignment method and a 3D pharmacophore model was constructed with five features of two hydrogen bond acceptors (A), one hydrogen bond donor (D), one aromatic ring (R), and one hydrophobic feature (H) simply represented by A2DRH. This pharmacophore was used to screen the phase database constructed from the Maybridge compound library and a total of 407 hit compounds were generated. These 407 compounds were then docked with the CatS structure (PDB ID: 3OVX) using RO5444101 as the standard and ligands with a gscore/docking score, molecular weight, PSA, and pharmacokinetics properties better than RO5444101 were considered further. These considerations yielded eight compounds with docking scores between −5.126 and −8.279 Kcal/mol. AW000699 (Figure 7) had a docking score of −8.277 kcal/mol lower than RO5444101 and better pharmacokinetic properties. It also interacted with amino acid residues at the S1, and S2 pockets and the catalytic center of CatS. However, its biological activities were below the reference compound. KM0787 (Figure 7) on the other hand with a docking score of −6.858, a molecular weight of 454.44 Da, PSA of −110.8 Å2 was the only compound with inhibitory values with an IC_50_ of 5000 nM. There was no report for the specific selectivity of KM0787 against other cathepsins members which have been established to be a standard in determining promising CatS-specific compounds. Apart from the improved pharmacokinetic properties (molecular weight, PSA) and CatS selectivity of KM0787, RO5444101 is a better and more potent CatS inhibitor [46].

### 4.7. Substrate-Based CatS Inhibitor

Substrate-based identification of CatS inhibitor through a blend of molecular modeling and synthesis was adopted by Galibert et al. and Wartenberg [55] to identify a CatS and K inhibitor. The strategy employed by Galibert and Wartenberg [55] to develop a stable competitive inhibitor of CatS was to modify substrate-like compounds into viable inhibitors. They derived four substrate-like compounds to check their inhibitory properties with CatS and K. The substrate-like compounds synthesized were two azapeptide compounds and two triazolepeptide compounds. They observed that the azapeptide substrate-derived inhibitors were more potent than the triazolepeptide on CatS and K when examined through in vitro screening and in vivo assay. The azapeptides have their IC_50_ in the nanomolar range whereas the triazoleleptides have their IC_50_ in the micromolar range. However, while it has been determined that potency is very important in identifying an inhibitor for CatS, equally important is the specificity of the inhibitor for CatS, to avoid the possibility of failure in clinical trials this was not well defined in their findings [55].

### 4.8. Other CatS Inhibitors

In 2013, Medivir AB resurrected its CatS inhibitor project and announced MIV-247 as a CatS inhibitor that has found use in neuropathic pain. MIV-247 is a highly selective, potent, and orally available CatS inhibitor [47]. It has a Ki of 2.1, 4.2, and 7.5 nM in humans (Table 1), mice, and cynomolgus monkeys, respectively [47]. Information on MIV-247 is very limited as it was only presented as an abstract at two international conferences on pain in 2014 in Buenos Aires and at Niece in 2015 [56].

Millipore-219393 (Table 1) is a CatS selective inhibitor that inhibits the expression of CatS by stimulating peroxisome proliferator-activated receptor-y (PPARy) to prevent pulmonary arterial hypertension because CatS overexpression promotes this disease [9]. Millipore-219393 has, however, been discontinued because a more potent and selective compound has been developed. Information on Millipore-219393 is very scant on the internet. However, some researchers have explored its use in the inhibition of CatS with very good outcomes. Researchers have explored and found use and importance for it in the treatment of pulmonary arterial hypertension and systemic lupus erythematosus (SLE) associated with pulmonary arterial hypertension with great success [9,57]. Apart from selectively inhibiting CatS, it is also able to stimulate PPARy to further inhibit CatS, which in turn improves the production and prevents the degradation of elastin which is responsible for the integrity of the basement membrane. The success recorded by Chang and Hsu [9] in their findings propelled Yen and Ho [57] to observe the effect of Millipore-219393 CatS inhibitor on the pulmonary arterial hypertension systemic lupus erythematosus (SLE) associated with pulmonary arterial Hypertension.

A highly potent and specific Cat-S inhibitor, RO5459072, was developed by Tato and Kumar [48]. Its inhibition of CatS in humans with an IC_50_ of 0.1 nM has been documented (Table 1). RO5459072 interacts with CatS through an irreversible covalent bond. Hargreaves and Daoudlarian [58] have explored the use of this specific inhibitor of the CatS enzyme in primary Sjögren syndrome. Also, Astellas Pharma Inc. developed a phenyldifluoromethyl-substituted prolineamide compound (ASP1617) (Table 1) which is a potent and selective CatS inhibitor for the management of autoimmune diseases such as systemic lupus erythematous [59]. ASP1617 has an IC_50_ of 4.6 nM in humans and 0.39 nM in mouse CatS enzyme and it has recently completed a phase I clinical trial (Table 2).

## 5. Development of CatS Inhibitors for Various Diseases

CatS expression has been associated with various diseases such as autoimmune disease, cancer, cardiovascular disease, pain, and a lot of other diseases. The expression of the CatS gene has been linked to tumor progression and it has been suggested that the expression of the CatS gene (ctss) is directly proportional to tumor progression in some types of cancer, especially brain cancer [60,61,62]. Cardiovascular disease and cancer are the leading causes of death worldwide and they are responsible for half of all death according to the 2019 WHO report. CatS has been found to be involved in the pathophysiology of these diseases and, most importantly, autoimmune diseases. So far, a few potential CatS inhibitors have been tested in some of these diseases. Some of these identified inhibitors are the target of different research to further understand their pharmacodynamics and pharmacokinetics in disease conditions.

### 5.1. Autoimmune Disease

RO5461111 is a competitive selective inhibitor of CatS developed by La Roche and has been explored for its use in a systemic autoimmune disease which is common in women, and systemic lupus erythematosus (SLE) [57]. Primary Sjögren syndrome is another form of autoimmune disease where CatS inhibition is beneficial [58]. A derivative of LY3000328 has also been explored for use in regulating the T-cell activity in bladder cancer [63]. CatS inhibition has found various uses in the management of autoimmune diseases.

### 5.2. Cancer

CatS inhibition has been the focus of research since its involvement in the initiation, progression, and outcomes of different diseases have been established [21]. The role of its inhibition in cancer immunotherapy has been explored through small molecule compounds with good pharmacokinetics properties to improve the limit of tumor expansion and the immunogenicity of the tumor environment [17,64,65]. CatS inhibition has been well studied in some tumor cells, brain tumors (glioblastoma) [60], oral cancer [66], bladder cancer [63], gastric cancer [67], breast cancer, colon cancer, follicular lymphoma, and non-Hodgkin lymphoma [68,69,70,71,72]. Zhang [60] investigated the effect of the inhibition of CatS in apoptosis and autophagy in brain tumors on the premise that the expression of CatS was very high in brain tumors (glioblastoma).

Since the identification of LY3000328 (CatS inhibitor), several researchers have adopted it to test its effectiveness as a CatS inhibitor in different disease models. Its inhibitory effect and pharmacological activities in oral squamous carcinoma were tested in vivo using male and female wild-type (WT) C57BL/6J mice and it was observed that LY3000328 was able to reduce tumor cell proliferation, thus improving disease outcome [4]. This CatS inhibitor has also been found to be useful in the regulation of the Bim protein associated with endoplasmic reticulum stress observed in renal carcinoma cells. In oxaliplatin-induced apoptosis in a renal cell line, pharmacological inhibition of CatS by LY3000328 increases the expression of the proapoptotic Bim protein through the AMPK signaling pathway [73]. A number of other researchers have found CatS inhibition to be useful in the management of different types of cancers.

### 5.3. Cardiovascular Disease

Only a few studies have been carried out on CatS inhibition as it relates to cardiovascular diseases. Peng and Liu [18] studied the inhibition of CatS in myocardial ischemia/reperfusion injury. In vivo research with CatS knockout mice buttresses the submission that CatS inhibition improves the disease state in collagen-induced arthritis, inflammation, and cancer [21]. The CatS inhibitor Millipore-219393 was identified to be effective in the management of pulmonary arterial hypertension. Its inhibition of CatS in systemic lupus erythematosus linked with PAH was explored by Yen and Ho) [57] in an in vivo model. Millipore-219393 was observed to inhibit CatS by stimulating peroxisome proliferator-activated receptor gamma (PPAR) in the animal lung. The effect was monitored in the lung, kidney, spleen, and liver but it was observed that CatS inhibition by Millipore-219393 was more effective in the lungs. The inhibitor was able to prevent abnormal remodeling of the heart muscle and pulmonary arterial wall. Additionally, RO5444101 has been used as an inhibitor of CatS in an in vivo study to modulate adipogenesis, inflammation, and liver cholesterol accumulation in an obesity model. CatS expression was seen to be elevated in the plasma, visceral adipose, and liver tissue of people consuming a high-fat diet, and 60 mg/mL of RO5444101 was found to be effective in managing obesity by reducing the animal weight, improving glucose tolerance and insulin tolerance significantly. It was also found to modulate cytokines, inducible nitric oxide synthase (iNOS), tumor necrotic factor-Alpha (TNF-α), interleukin-1beta (IL-1β), and interleukin-6 (IL-6) which were elevated by adiposity. However, Arg1 and IL-10 levels were increased with the presence of RO5444101 as a CatS inhibitor [74].

### 5.4. Pain

Xu and Wang [75] explored the neuroprotective effect of LHVS (an irreversible, potent non-selective CatS inhibitor) in post-traumatic brain injury in mice, and the upregulation or the increased expression of CatS post-brain injury was reported. They also established that the presence of endogenous CatS inhibitors is not enough to attenuate the effect of increased CatS expression in the pathological phase. Hence, the presence of LHVS helps to alleviate the effect of the increased expression of CatS in cerebral edema, and this is believed to be achieved by the regulation of the inflammatory process. Hewitt and Pitcher [47] have explored the use of MIV-247 together with gabapentin or pregabalin for neuropathic pain management through the CatS pathway. In their findings, they discovered that using MIV-247 together with either pregabalin or gabapentin increases the drugs’ efficacy without any form of alteration to the pharmacokinetics of both drugs (pregabalin and gabapentin), especially its specificity for the CatS enzyme.

## 6. Prospective Areas for Identifying CatS Inhibitors

Plants have been used since antiquity for the treatment and management of diseases. Plant secondary metabolites are good sources of pharmacological compounds or drugs. It has been reported that plants rich in flavan-3-ols, flavonoids, chalcones, and triterpenes are good in the management of cardiovascular disease and other diseases [76]. Plants rich in antioxidants have also been proven to be effective in disease management, especially in the mopping up of free radicals. Also, the pentacyclic triterpenoids which include asiaticoside, asiatic acid, madecassoside, madecassic acid, and asiaticoside-D have been indicated for action against several diseases including cardiovascular diseases [38,77,78]. Vidal-Albalat and González (2016) have explored the use of natural compounds as inhibitors for cathepsin and certain natural source compounds like Miraziridine A, Gallinamide A, Symplostatin 4, Panduratin A, Nicolaioidesin C, and others have been identified as inhibitors for CatK, CatL, and CatV [79]. A natural product Asperphenamate and its derivatives (B-2a) have also been explored for its inhibitory activity against cysteine protease especially CatL with very promising outcomes [80]. This outcome propelled Huang et al. (2023) to explore the CatS inhibitory activities of the Asperphenamate derivative since the 2 cysteine proteas have similar structural components [49]. This resulted in the identification of ASPER-29 which is a dual CtaS and CatL inhibitor. It would be interesting to explore the use of plant secondary metabolites in the management of disease as it concerns the specific inhibition of CatS.

## 7. Conclusions

In recent times there has been an increased interest in CatS inhibitors, especially because of the findings of their importance, benefit, and involvement in the pathophysiology of cardiovascular disease and cancer. The focus of current research has improved greatly from what it used to be in the early 2000s, due to the advances in technology and high throughput screening. Research has focused on advancement in technology to develop an inhibitor for CatS. Also, in the past, companies were the only ones with the financial ability to develop new drugs, but it has been observed in recent times that research groups are also working on CatS inhibitors and adopting different molecular mechanism methods. It is important to identify a non-covalent selective and potent cathepsin inhibitor but the best method to adopt is to focus on the interaction with the amino acid residue at the S2 and S3 to achieve this. One of the most successful methods in the identification of CatS inhibitors is the NMR Fragment Screening method. It has been able to propose a lead compound with excellent selectivity which interacts with the amino acid residue at the S2 and S3 pocket of the CatS enzyme, and this makes the compound more selective. Focusing on identifying an inhibitor for CatS from natural compounds and screening cysteine protease inhibitors to identify an inhibitor with good pharmacokinetic and pharmacodynamics properties will also be worthy ventures as there are limited data to this effect.

## Figures and Tables

**Table 1:** List of CatS inhibitors and their IC_50_ (nM).

S/N	Compound Name	IC_50_ nM	CatS Selectivity	References
1	Compound **2**	3.3	Yes	[34]
2	Compound **3**	1.2	Yes	[35]
3	Compound **5**	60	Yes	[38]
4	compound **9**	45	Yes	[44]
5	Compound **10**	0.7	Yes	[40]
6	LY3000328	7.7	Yes	[42]
7	RO5444101	0.2	Yes	[45]
8	KM0787	5000	No report	[46]
9	MIV-247	1	No report	[47]
10	Millipore-219393	not available	not available	[9]
11	RO5459072	not available	not available	[48]
12	ASP1617 disuccinate	4.6	Yes	
13	ASPER-29 (Asperphenamate derivative)	1790	No	[49]

**Table 2:** A list of CatS drugs at different stages of clinical trials (Adopted from https://clinicaltrials.gov) (Accessed on 10 June 2024) [54].

S/N	Drug Name	Company	Indication	Phase	Clinical Trial Identifier
1	VBY-036	Virobay Inc., Menlo Park, CA, USA	Nerve pain	Phase I	NCT01911637
2	RWJ-445380	Johnson & Johnson Pharmaceutical Research and Development, LLC, Raritan, NJ, USA	Rheumatoid Arthritis	Phase II	NCT00425321
3	VBY-891	Virobay Inc., Menlo Park, CA, USA and LEO Pharma Ballerup, Ballerup, Denmark	Psoriasis (inflammatory autoimmune disease)	Phase I	NCT01947738
4	Not Provided	Children’s Hospital of Orange County, Orange, CA, USA and Ultragenyx Pharmaceutical Inc., Novato, CA, USA	Mucopolysaccharidoses	Recruiting for Phase I	NCT05063435
5	IPHAAB	Medical University of Vienna and Austrian Federal Ministry of Defense and Sports, Vienna, Austria	Not indicated	Not indicated	NCT02097199
6	RO5459072	Hoffmann-La Roche, Basel, Switzerland	Celiac Disease	Phase I	NCT02679014
7	LY3000328	Eli Lilly and Company, Indianapolis, IN, USA	Not indicated (abdominal aortic aneurysm)	Phase I	NCT01515358
8	LIPOGAIN	Uppsala University, Uppsala, Sweden	Weight Gain	Not Applicable	NCT01427140
9	Vorinostat	Boston University and Merck Sharp & Dohme LLC, Rahway, NJ, USA	Mycosis Fungoides	Withdrawn	NCT01801670
10	ASP1617disuccinate	Astellas Pharma Global Development, Inc., Northbrook, IL, USA	Autoimmune diseases (systemic lupus erythematosus)	Phase I	NCT04077879
11	LIPOGAIN	Uppsala University, Uppsala, Sweden	Weight Gain	Not Applicable	NCT01427140

## Data Availability

All data are provided in this article.
